# Analysis of Two CDKN2B-AS Polymorphisms in Relation to Coronary Artery Disease Patients in North of Iran

**Published:** 2017-01-17

**Authors:** Maryam Mafi Golchin, Sayyed Mohammad Hossein Ghaderian, Haleh Akhavan-Niaki, Rozita Jalalian, Laleh Heidari, Seyed Alireza Salami

**Affiliations:** 1 *Department of Genetics, Faculty of Medicine, Babol University of Medical Sciences, Babol, Iran. *; 2 *Department of Medical Genetics, Faculty of Medicine, Shahid Beheshti University of Medical Sciences, Tehran, Iran.*; 3 *Cellular and Molecular Biology Research Center, Babol University of Medical Sciences, Babol, Iran.*; 4 *Cardiovascular Research Center, Mazandaran University of Medical Sciences, Sari, Iran.*; 5 *Department of Biotechnology, University of Tehran, Tehran, Iran.*

**Keywords:** Single nucleotide polymorphism, risk factor, coronary artery disease, *CDKN2B-AS*, Iranian

## Abstract

Coronary artery disease (CAD) including myocardial infarction (MI) as its complication, is one of the most common heart diseases worldwide and also in Iran, with extremely elevated mortality. CAD is a multifactorial disorder. Twin and family studies at different loci have demonstrated that genetic factors have an important role in the progression of CAD. Many studies have reported a significant association of *CDKN2B-AS*, also known as *ANRIL* which is located within the p15, p16, p14 gene cluster at 9p21 locus, with cardiovascular diseases as well as many other diseases like diabetes and cancers. This study investigated two polymorphisms rs10757274 and rs1333042 of *CDKN2B-AS* gene at 9p21 locus. 205 subjects, comprising 102 controls and 103 CAD patients were genotyped by TaqMan probe real time PCR technique and haplotypes were examined. This study confirmed the association of rs10757274 variants with CAD in Iranian patients (P= 0.003) but genotype and allele distributions of CAD and control groups showed no significant association for the rs1333042. However, frequency of the [G;G] haplotype of these two SNPs was significantly higher in CAD group (P= 0.0002, Odds Ratio = 3.1, 95% CI = 1.7-5.7). Our finding suggests that [G; G] haplotype of rs10757274 and rs1333042 may be considered as a genetic risk factor for susceptibility to CAD in Iranian patients.

Coronary artery disease (CAD) including myocardial infarction (MI) as its complication, is one of the leading causes of death and disability worldwide (1). CAD and MI are complex multifactorial and polygenic disorders, which are believed to be caused by both genetic and environmental factors (2, 3). Although the most important determining factors are age (4), hypertension (5), family history (6, 7), and cholesterol level (8), however twin and family studies at different loci have demonstrated that genetic factors have important role in the progression of CAD and have been defined as an important risk for the pathogenesis of CAD and MI (2, 9).

From 2007, independent studies using single nucleotide polymorphism (SNP) arrays have shown that chromosome 9p21 is associated with increased risk of CAD and MI (10-13). *CDKN2B-AS*, also known as *ANRIL* is a long non-coding RNA which is located within the p15/*CDKN2B*-p16/*CDKN2A*-p14/*ARF* gene cluster at 9p21 locus (14). Multiple alternatively spliced transcript variants have been generated from this gene, and all of them are long non-coding RNAs (15-17). Many studies have reported the significant association of *CDKN2B-AS* with cardiovascular diseases and also many other diseases like diabetes and cancers (17-20). Moreover, *CDKN2B-AS* is expressed in tissues and cell types that are affected by atherosclerosis (18) and associated with formation of atherosclerotic plaques (21).

Over the past years, several genome- wide association studies have identified a lot of variants at 9p21 locus which were associated with risk of CAD and MI (22-25). Most of the studies have been performed in north American and north European (22-25) or in East Asian populations (26-28) and Middle East studies have been accomplished within small size populations (29, 30). On the other hand, it was recently reported that the prevalence of CAD and its risk factors in Iranian population is very high and even more than Western countries (14). Thus, more studies are necessary in middle East region including Iran.

In the present study we genotyped two SNPs: rs10757274 and rs1333042 at 9p21 locus, selected on the basis of previously reported associated variants in GWAS with CAD, and also performed haplotype analysis of those variants in North Iranian patients.

## Materials and methods

**Patients and study protocol**

The study population comprised 103 CAD patients and 102 control subjects (49% males and 51% females, mean age 59.08±8.78 vs 61.14± 11.07 years). All samples were collected from Rohani hospital in Babol and Fatemeh Zahra clinic of Mazandaran heart center in Sari (north of Iran) during 2014. The study was designed according to world health organization criteria and the diagnostic criteria for CAD included 50% luminal narrowing in at least one vessel by coronary angiography or myocardial infarction.

All control subjects were selected from both gender over the age of 40 years and showed no signs of CAD, and other risk factors such as hypertension, high cholesterol level, high body mass index (BMI), diabetes mellitus and cancers. Hypertension was defined as systolic blood pressure (SBP) of 140 mm Hg or above, or diastolic blood pressure (DBP) of 90 mm Hg or above. Diabetes was defined as fasting blood sugar of 126 mg/dl or above. The controls had completely normal coronary arteries with no evidence of common risk factors of CAD. The clinical characteristics of both CAD cases and controls are shown in Table 1. This study was approved by the ethical committee of Babol University of Medical Sciences. At the time of sampling, a complete clinical history was collected from all participants and written informed consent was obtained from all of them.

**Genotyping**

Genomic DNA was extracted from venous blood (5ml) using a commercially available kit (Roche, Germany). The quality of the extracted DNAs was analyzed by gel electrophoresis and was confirmed by spectrophotometry (Nanodrop1000, Thermo Fisher Scientific, Wilmington, DE, USA). Primer-probe sets were designed by ABI (Applied Bio systems, Foster City, CA, USA). Genotyping for the rs10757274A>G (Assay ID C__26505812_10) and rs1333042A>G(Assay ID C__1754675_10) in *ANRIL* gene was performed using TaqMan probe real time PCR (LightCycler 96, Roche, Germany).

**Statistical Analysis**

Statistical analysis of the data was carried out by SPSS, version 18. Normally distributed data were expressed as means± standard deviation (SD) or median and qualitative variables were expressed as n (%). The statistical difference in allelic and genotype frequencies between patients and controls were compared using the chi- square test and P value<0.05 was considered as statistically significant and for haplotype analyzing of the data, SNP Stats software which has online version as well (http:// bioinfo.iconcologia.net/ snpstats) was used.

## Results

**Clinical characteristics**

Detailed information of CAD patients and controls is provided in Table 1. The mean age values, sex, body mass index (BMI), height and weight were homogeneous in the studied subjects. SBP, DBP, HDL cholesterol and smoking had significantly better levels in healthy control subjects compared with CAD patients. However, obesity, hypertension, total cholesterol, FBS, LDL-cholesterol and diabetes were significantly higher in the CAD group (P<0.05 for each variable; Table 1).

The genotype and allele frequencies of rs10757274 and rs1333042 were found to be in Hardy-Weinberg equilibrium (Table 2). For rs10757274, the frequencies of AA, AG and GG genotypes in CAD group were 15.5%, 45.6% and 38.9%, respectively versus 28.4%, 50% and 21.6%, respectively in control group, and allele G distribution showed significant association with CAD (61.7% in patients versus 46.4% in controls) (P= 0.002).

**Table 1:** Clinical features of CAD and healthy subjects

**Base line characteristics**	**CAD patients** **(n=103)**	**Controls ** **(n=102)**	**P-value**
Age (years)	59.08±8.78	61.14±11.07	0.14
Male (%)	43(49.4%)	44(50.6 %)	0.50
Height (cm)	161.02±9.81	161.39±8.73	0.777
Weight (kg)	70.03±13.94	69.77±12.77	0.893
BMI (kg/m²)	26.97±4.82	26.33±4.61	0.333
Obesity (%)	28.2%	12.6%	0.006
SBP (mm Hg)	137.25±26.58	122.27±18.83	0.000
DBP (mm Hg)	83.77±12.83	73.20±13.05	0.000
Hypertension ^a^ (%)	54.36%	32%	0.005
TC (mg/dl)	171.19±32.24	192.72±27.37	0.000
TG (mg/dl)	152.57±67.55	181.10±53.73	0.001
FBS (mg/dl)	140.35±64.49	122.19±39.62	0.016
HDL (mg/dl)	38.94±7.47	41.80±5.83	0.002
LDL (mg/dl)	101.02±23.49	115.1±23.26	0.000
Smoking^b^ (%)	43.7%	7.8%	0.000
Diabetes ^c^	49%	33%	0.012

Data presented as mean±SD, n (%) of cases or median.

a Systolic blood pressure above 140 mmHg, and/or diastolic blood pressure above 90 mmHg, and/or history of hypertension, and/or current anti-hypertensive medication.

b Ceased smoking >1 year prior to enrolment and

c diabetes (fasting blood sugar above 126 mg/dL) BMI: body mass index; SBP: systolic blood pressure; DBP: diastolic blood pressure; TC: total cholesterol; TG: triglycerides; FBS: fasting blood sugar; HDL: high-density lipoprotein cholesterol; LDL: low-density lipoprotein cholesterol.

**Table 2:** Genotypes and alleles distribution of rs10757274 and rs1333042

**P-value**	**OR (95% CI)**	**Controls **	**CAD patients **	
	**-**	**N=102(%)**	**N=103(%)**	**rs10757274**
	1.00 (reference)	29(28.4%)	16(15.5%)	AA
0.167	1.67(0.80 – 3.45)	51(50.0%)	47(45.6%)	AG
0.003	3.29 (1.47-7.34)	22(21.6%)	40(38.9%)	GG
-	reference	109(53.4%)	79(38.3%)	Allele A
0.002	1.8(1.24-2.73)	95(46.4%)	127(61.7%)	Allele G
-	-	N=102	N=103	rs1333042
	1.00 (reference)	12(11.8%)	19(18.4%)	AA
0.278	0.63(0.27-1.44)	46(45.1%)	46(44.7%)	AG
0.158	0.54(0.23- 1.26)	44(43.1%)	38(36.9%)	GG
-	reference	70(34.3%)	84(40.7%)	Allele A
0.177	0.75(0.50-1.13)	134(65.7%)	122(59.3%)	Allele G

**Table 3:** Distribution of 9p21 haplotypes [rs10757274 A/G; rs1333042 A/G

**rs10757274**	**rs1333042**	**Haplotype**	**CAD(n=103)**	**Control(n=102)**	**OR(95%CI)**	**P value**
**A**	A	AA	23%(24)	53%(45)	1.00 (reference)	_
**G**	A	GA	15%(16)	29%(30)	1.1(0.5-2.63)	0.66
**A**	G	AG	5%(6)	4%(5)	2.88(0.7-11.7)	0.13
**G**	G	GG	78%(80)	57%(58)	3.1(1.7-5.7)	0.0002

Observed frequencies in rs1333042 for CAD genotypes included 18.4% (AA), 44.7% (AG), and 36.9% (GG). On the other hand, frequencies for controls were, 11.8% (AA), 45.1% (AG), and 43.1% (GG) (Table 2). The association between the frequencies of the two alleles and CAD occurrence remained insignificant (P=0.17, Odds ratio=1.31, 95% CI= 0.88-1.96)

The distribution of rs10757274 and rs1333042 haplotypes at 9p21 is summarized in Table 3. The frequency of [G; G] haplotype was 78% in CAD versus 57% in control group (P= 0.0002, OR= 3.1, 95% CI: 1.7-5.7).

## Discussion

Cardiovascular disease is considered as one of the most leading causes of death worldwide (1) and world health organization estimated that by 2030 more than 23 million people will die annually from this disease. It was recently reported that the prevalence of CAD and its risk factors in Iranian population is very high and even more than Western countries (14).

A number of studies investigated the demographic and clinical factors, associated with CAD. In the present study, after statistical analyzes and matching differences between means of variances of risk factors among different groups (age and sex), significant differences for some CAD risk factors including obesity, hypertension, total cholesterol, triglycerides, FBS, HDL, LDL and diabetes were observed (P<0.05), confirming the results of previous reports including those of Iranian population (31, 32).

For decades, several genome wide association studies focused on genes and genetic markers associated with an increased risk of CAD and MI. Many of these studies have identified a number of variants at 9p21 locus as having a relation with risk of CAD and MI development (8, 9, 11).

The rs10757274 is one of the most intensively examined polymorphisms of *CDKN2B-AS* gene at 9p21 locus. It was initially identified by McPherson et al. and subsequent studies confirmed its association with CAD and MI (22, 33-37). Also, a recent study by Nawaz et al. in a small Pakistani population has revealed a strong association of the GG genotype with CAD (OR:9.603; 95% CI: 5.746-16.05) (29). On the other hand, the results of the study of Dehghan et al. in 2008 about risk of congenital heart disease and MI in a large population based study in Rotterdam showed no association between the above- mentioned genotype and CAD (23).

In the present study, investigation of the rs10757274 showed a significant difference of allele G distribution between patients (61.7%) and controls (46.4%) (P=0.002). Hence, our results were in accordance with previous studies (25-29) and also a recent study of rs10757274 in South-West Iran (30) presenting G allele as CAD risk factor.

Also, several researches showed that rs1333042 which is located in *CDKN2B-AS* gene, has association with CAD and MI (38-41). Matarin et al. investigated the ischemic stroke and heart disease in North Americans and reported a significant association of dominant model but the risk allele G showed an opposite association, being protective against disease with P= 0.042 (38). In the present study, the prevalence of GG genotype of rs1333042, and the frequency of G allele were not statistically different among studied groups (P= 0.17) but G allele frequency was higher in CAD patients which is in conflict with Matarin et al.’s study. However, in a recent study by Kurki et al. which was performed on Finnish population with intracranial aneurysms (IA), among 17 SNPs, only 4 including rs1333042 showed significant association with IA (OR =1.31, CI 1.21–1.42, P= 1.8×10^−11^) (39). Correspondingly, many indepe-ndent replication studies are needed to validate phenotype association of these important SNPs in different populations. One reason for this controversy between different studies may be the heterogeneity of risk factors and complexities of their role in CAD and polygenic diseases in general. Each genetic polymorphism may have only a small effect.

Our main goal was to replicate the association between rs10757274 and rs1333042 in *CDKN2B-AS* gene at 9p21 locus with CAD. As haplotypes are more powerful for detecting susceptibility of disease than individual polymorphisms, we also analyzed the effect of different haplotypes of these two SNPs on disease development and found that [G; G] haplotype was associated with CAD development (P= 0.0002).

Moreover, one of the limitations of this study is the sample size which may not be large enough to allow such differences to appear. The other limiting factor of the present study is that it was performed on a small part of Iranian population (North of Iran), because independent studies in Iran showed genetic variability (42).

In conclusion, the association of rs10757274 variants with CAD in Iranian patients (P =0.003) was confirmed by the present study but genotype and allele distributions of CAD and control groups showed no significant association for the rs1333042 polymorphism.

However, the frequency of [G; G] haplotype of these two SNPs was significantly higher in CAD group (P = 0.0002).

Our finding suggests that [G; G] haplotype of rs10757274 and rs1333042 may be considered as a genetic risk factor for susceptibility to CAD in North Iranian patients. These data are the beginning of a comprehensive association study of 9p21 haplotype polymorphisms with CAD in Iranian population, although consistent association studies in larger sample sizes are necessary to replicate our findings.

## Conflict of interest

The authors declared no conflict of interest.
